# Quercetin Protects H9c2 Cardiomyocytes against Oxygen-Glucose Deprivation/Reoxygenation-Induced Oxidative Stress and Mitochondrial Apoptosis by Regulating the ERK1/2/DRP1 Signaling Pathway

**DOI:** 10.1155/2021/7522175

**Published:** 2021-08-19

**Authors:** Fen Li, Dongsheng Li, Shifan Tang, Jianguang Liu, Jie Yan, Haifeng Chen, Xisheng Yan

**Affiliations:** ^1^Department of Neurology, Wuhan Third Hospital & Tongren Hospital of Wuhan University, Wuhan 430074, China; ^2^Department of Cardiology, Wuhan Third Hospital & Tongren Hospital of Wuhan University, Wuhan 430074, China; ^3^Department of Forensic Science, Changsha 410013, China; ^4^Department of Clinical Medicine, Jianghan University, Wuhan 430056, China

## Abstract

Reperfusion of blood flow during ischemic myocardium resuscitation induces ischemia/reperfusion (I/R) injury. Oxidative stress has been identified as a major cause in this process. Quercetin (QCT) is a member of the flavonoid family that exerts antioxidant effects. The aim of this study was to investigate the preventive effects of QCT on I/R injury and its underlying mechanism. To this end, H9c2 cardiomyocytes were treated with different concentrations of QCT (10, 20, and 40 *μ*M) and subsequently subjected to oxygen-glucose deprivation/reperfusion (OGD/R) administration. The results indicated that OGD/R-induced oxidative stress, apoptosis, and mitochondrial dysfunction in H9c2 cardiomyocytes were aggravated following 40 *μ*M QCT treatment and alleviated following the administration of 10 and 20 *μ*M QCT prior to OGD/R treatment. In addition, OGD/R treatment inactivated ERK1/2 signaling activation. The effect was mitigated using 10 and 20 *μ*M QCT prior to OGD/R treatment. In conclusion, these results suggested that low concentrations of QCT might alleviate I/R injury by suppressing oxidative stress and improving mitochondrial function through the regulation of ERK1/2-DRP1 signaling, providing a potential candidate for I/R injury prevention.

## 1. Introduction

Cardiovascular diseases are the primary cause of human mortality worldwide, leading to more than 17 million deaths annually and accounting for 31% of global mortality [1, 2]. Ischemic heart disease is a major subset of cardiovascular diseases that results in hypoxia and cardiomyocyte death [3]. Reperfusion of blood flow is the only effective therapeutic strategy in clinical therapy for ischemic or hypoxic myocardium resuscitation [4, 5]. Unfortunately, reperfusion induces a series of complicated adverse reactions known as ischemia/reperfusion (I/R) injury, leading to secondary damage [6, 7]. Excessive reactive oxygen species (ROS) generation induces oxidative stress, and calcium ion overload has been identified as the major process responsible for I/R injury [8, 9]. Excessive ROS generation leads to the opening of the mitochondrial permeability transition pore, lipid peroxidation, and DNA damage, thereby causing apoptosis [10, 11]. Thus, strategies that prevent or alleviate oxidative stress response and cardiomyocyte apoptosis are advisable for the treatment of myocardial I/R damage.

Quercetin (QCT) is a member of the flavonoid family and can be extracted from vegetables, tea, fruits, wine, and various nutritious products [12]. Many pharmacological actions of QCT have been identified, including cardiovascular protective [12], anticancer [13, 14], anti-inflammatory [15], and antioxidant effects [16]. Panchal et al. showed that QCT mitigated cardiovascular remodeling, abdominal obesity, and nonalcoholic fatty liver disease in diet-induced metabolic syndrome in rats by attenuating inflammation and oxidative stress [17]. In addition, QCT alleviated DOX-induced toxicity in cardiomyocytes by promoting mitochondrial function and attenuating oxidative stress [18]. QCT demonstrated cardioprotective effects under I/R damage, which may be due to its effect on Src kinase dephosphorylation and STAT3 kinase-mediated inflammatory response blocking [19]. However, the preventive effect of QCT against myocardial I/R damage and its underlying mechanisms needs to be elucidated.

Based on these findings, we hypothesized that QCT can act as a candidate for myocardial I/R damage prevention, and its mechanism may be associated with oxidative stress and mitochondrial function modulation. To address this, H9c2 cardiomyocytes were treated with different concentrations of QCT, and N-acetylcysteine (NAC), a ROS inhibitor, was selected as a positive control. The cells were then treated with oxygen-glucose deprivation/reperfusion (OGD/R). The oxidative stress response and mitochondrial function of H9c2 cells were evaluated.

## 2. Materials and Methods

### 2.1. Cell Culture and Treatment

The rat myocardial cell line H9c2, supplied by Shanghai Institutes for Biological Sciences, Chinese Academy of Sciences, was cultured in Dulbecco's Modified Eagle's Medium (DMEM, HyClone, Logan, UT, USA) supplemented with 10% fetal bovine serum (FBS, Gibco, Waltham, MA, USA) and incubated at 37°C with 5% CO_2_ and 95% air. After the confluence reached 80%–90%, H9c2 cells were treated for 24 h with different concentrations (10, 20, 40, 80, and 160 *μ*M) of quercetin (QCT, Aladdin) and subsequently cultured for 6 h in glucose-free DMEM (TBD) at 37°C with 5% CO_2_, 5% O_2_, and 90% N_2_. Thereafter, the cells were cultured for 24 h in DMEM (HyClone) supplemented with 10% FBS (Gibco) and maintained at 37°C with 5% CO_2_ and 95% air. H9c2 cells in the OGD/R model group were cultured for 6 h in glucose-free DMEM (TBD) at 37°C with 5% CO_2_, 5% O_2_, and 90% N_2_ and for 24 h in DMEM (HyClone) supplemented with 10% FBS (Gibco) at 37°C with 5% CO_2_ and 95% air without QCT treatment. Cells in the control group were cultured in DMEM (HyClone) supplemented with 10% FBS (Gibco) and maintained at 37°C with 5% CO_2_ and 95% air. Cells in the NAC group were treated for 24 h with 10 mM NAC (Aladdin), a reactive oxygen species (ROS) inhibitor, and subsequently cultured for 6 h in glucose-free DMEM (TBD) at 37°C with 5% CO_2_, 5% O_2_, and 90% N_2_. Subsequently, the cells were cultured for 24 h in DMEM (HyClone) supplemented with 10% FBS (Gibco) and maintained at 37°C with 5% CO_2_ and 95% air. Proliferation, apoptosis, oxidative responses, and cellular mitochondrial function were evaluated.

### 2.2. Cell Counting Kit-8

Cell Counting Kit-8 (CCK-8) was used to detect the proliferation of H9c2 cells. 100 *μ*L of harvested H9c2 cells (3 × 103 cells per well) was seeded into 96-well plates in overnight. After different treatments, the cells were cultured for 4 h with additional 10 *μ*L of CCK-8 solution (Solarbio, Beijing, China) and subjected to a microplate reader (Allsheng, Hangzhou, China) to detect the absorbance at 450 nm.

### 2.3. Malondialdehyde, Superoxide Dismutase, Endothelial Nitric Oxide Synthase, and Adenosine Triphosphate Levels in H9c2 Cells

The levels of malondialdehyde (MDA, cat. no. A003-1-1, Nanjing Jiancheng Bioengineering Institute, Nanjing, China), total superoxide dismutase (SOD, cat. no. A001-3-2, Nanjing Jiancheng Bioengineering Institute), and adenosine triphosphate (ATP, cat. no. A095-1, Nanjing Jiancheng Bioengineering Institute) were determined by Nanjing Jiancheng Kit. Among them in the determination of malondialdehyde experiments, add the standard with the concentration of 10 nmol/ml to the standard tube, absolute ethanol into the blank tube, and samples into the determination tube and control tube and finally add reagent I into each tube. After fully mixing, add 3 ml reagent II and 1 ml reagent III to each tube (except the control tube) and finally add 50% glacial acetic acid to the control tube. All the tubes were mixed, bathed at 95°C for 40 minutes, and then fixed at 3500–4000 RPM for 10 minutes. The absorbance of the supernatant was measured at 532 nm and 1 cm diameter. In the experiment of superoxide dismutase, add 20* μ*L sample to the determination well and the determination of the blank well, add 20* μ*L distilled water to the control well and the control blank well, add 20* μ*L working liquid to the control well and the determination of the blank well, and add 20* μ*L diluent to the control blank well and the determination of the blank well. Finally, 200 *μ*L of substrate application solution was added to each well, the tubes were mixed and incubated at 37°C for 20 min, and the absorbance was read at 450 nm is carried out according to the instructions of Nanjing Jiancheng ELISA kit. Its principle is to bind antigen or antibody to the surface of solid-phase carrier and maintain its immune activity. Antigen or antibody is connected with a certain enzyme. Enzyme marks antigen or antibody. Enzyme marks antigen or antibody, retains its immune activity, but also retains enzyme activity. During the measurement, the tested specimen (the antibody or antigen measured in it) and the enzyme-labeled antigen or antibody react with the antigen or antibody on the surface of the solid-phase carrier in different steps. The antigen-antibody complex formed on the solid-phase carrier was separated from other substances by the washing method. Finally, the amount of the enzyme combined on the solid-phase carrier was proportional to the amount of tested substances in the sample. After adding the substrate of the enzyme reaction, the substrate was catalyzed by the enzyme into colored products, and the amount of the product was directly related to the amount of the substance tested in the specimen, so it can be qualitative or quantitative analysis according to the depth of the color reaction.

### 2.4. Flow Cytometry Assay

Flow cytometry was performed to detect reactive oxygen species (ROS) formation, apoptosis, and mitochondrial membrane potential (MMP) in H9c2 cells. For the ROS generation assay, 1 × 106 H9c2 cells in each group were resuspended in 1 mL of diluted DCFH-DA and maintained at 37°C for 20 min. Thereafter, the cells were treated with 500 *μ*L phosphate-buffered saline (PBS) and analyzed with flow cytometry (ACEA Biosciences, San Diego, CA, USA). For the apoptosis assay, 1 × 106 H9c2 cells in each group were centrifuged at 400 × *g* at 4°C for 5 min, resuspended in 200 *μ*L PBS, and stained for 30 min with 10 *μ*L annexin V-fluorescein isothiocyanate (FITC) and 10 *μ*L propidium iodide (PI) in the dark at 4°C. After 300 *μ*L PBS was added, the cells were analyzed with flow cytometry (ACEA Biosciences). For the MMP assay, 1 × 106 H9c2 cells in each group were resuspended in 500 *μ*L DMEM and cultured for 20 min with JC-1 solution (Beyotime) at 37°C. Thereafter, the cells were centrifuged at 400 × *g* at 4°C for 3 min and resuspended in 1 mL of JC-1 solution. The cells were then centrifuged at 400 × *g* at 4°C for 3 min, resuspended in 400 *μ*L of JC-1 solution, and analyzed with flow cytometry (ACEA Biosciences).

### 2.5. Transmission Electron Microscope

Transmission electron microscopy (TEM) was performed to observe the ultrastructure of the mitochondria in H9c2 cells. A total of 1 × 107 cells in each group were fixed for 30 min in 2 mL of 2.5% glutaraldehyde at 4°C and then fixed for 1 h in 1% osmic acid. After dehydration, soaking, and embedding, ultrathin sections (∼60 nm) were obtained and stained with uranyl acetate for 20 min and lead citrate for 15 min in the dark. The ultrastructure of the mitochondria was observed using a transmission electron microscope (Hitachi, Tokyo, Japan).

### 2.6. Western Blot Assay

Western blotting was performed to detect apoptosis-related, mitochondrial function-related, and extracellular signal-regulated kinase (ERK) protein expression in H9c2 cells. Total protein was extracted from H9c2 cells using radioimmunoprecipitation assay lysis buffer (Solarbio), and protein concentration was quantified using a bicinchoninic acid assay kit (Solarbio). Twenty micrograms of proteins from each group were separated by sodium dodecyl sulfate-polyacrylamide gel electrophoresis and transferred onto polyvinylidene fluoride membranes. After blocking with 5% skim milk, the membranes were incubated for 1 h with primary antibodies against dynamin-related protein 1 (DRP1), mitofusin 2 (MFN2), phosphorylation (p)-DRP1-S637, B-cell lymphoma 2 (Bcl-2), Bcl-2-associated X (Bax), cleaved caspase-3, ERK1/2, p-ERK1/2, and *β*-actin (internal reference), followed by 1 h of incubation with the goat anti-rabbit IgG secondary antibody. All antibodies were purchased from Bioswamp, except for cleaved caspase-3 (Abcam, Cambridge, UK), p-ERK1/2 (Abcam), and p-DRP1 (CST).

### 2.7. Statistical Analysis

Data are presented as the mean ± standard deviation (SD). Differences among groups were analyzed using one-way analysis of variance (ANOVA) followed by Tukey's test. Statistical significance was set at *P* < 0.05.

## 3. Results

### 3.1. Low Concentrations of QCT Promoted Proliferation and Inhibited Oxidative Stress Response in OGD/R-Induced H9c2 Cells

**Figure 1:**
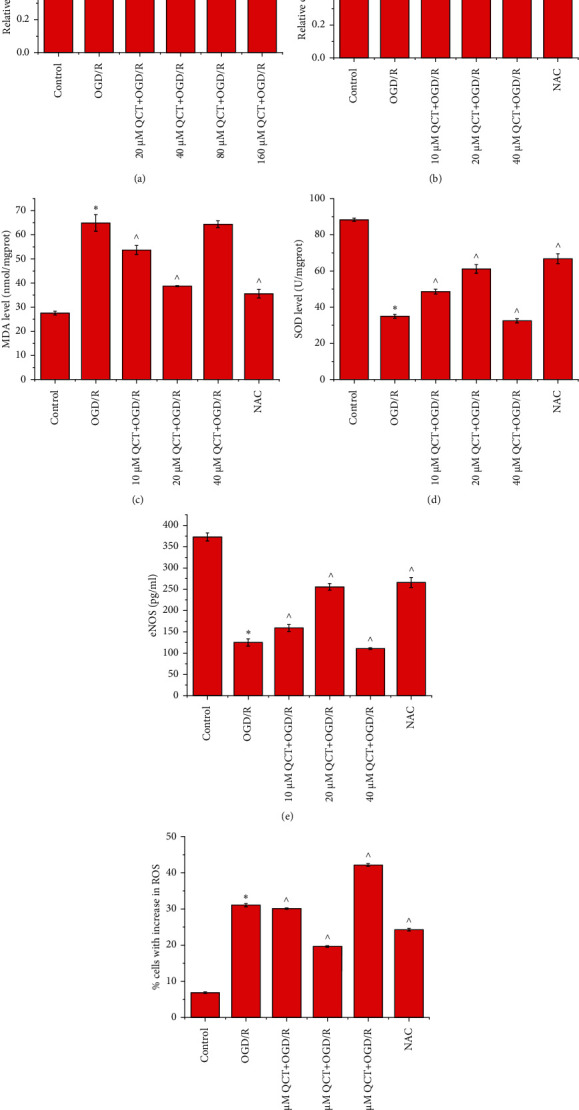
Low concentration of QCT promotes proliferation and inhibits oxidative stress response in OGD/R-induced H9c2 cells. The (a, b) viability, (c) MDA level, (d) SOD level, (e) eNOS level, and (f) proportion of H9c2 cells with increased ROS. Cells were cultured with different concentrations of QCT or NAC and subsequently cultured for 6 h in glucose-free DMEM (TBD) at 37°C with 5% CO_2_, 5% O_2_, and 90% N_2_; thereafter, the cells were cultured for 24 h in DMEM (HyClone) supplemented with 10% FBS (Gibco) and maintained at 37°C with 5% CO_2_ and 95% air. Data were presented as the mean ± SD, *n* = 3, ^*∗*^*p* < 0.05 vs. the control group, and ^*p* < 0.05 vs. the OGD/R group.

The CCK-8 assay showed that OGD/R treatment inhibited the proliferation of H9c2 cells. A low concentration (20 *μ*M) of QCT weakened this effect, while high concentrations (more than 40 *μ*M) of QCT strengthened it prior to OGD/R treatment (Figure 1(a)), indicating the preventive effect of low concentrations of QCT on OGD/R-induced damage in H9c2 cells. QCT concentrations of 10, 20, and 40 *μ*M were chosen for subsequent experiments to investigate the specific effects of QCT on OGD/R-induced damage. The results indicated that OGD/R treatment decreased the proliferation (Figure 1(b)), SOD (Figure 1(d)), and eNOS (Figure 1(e)) levels and increased the MDA level (Figure 1(c)) and the proportion of cells with increased ROS (Figure 1(f)). The effects of OGD/R were aggravated with 40 *μ*M QCT treatment and mitigated with 10 and 20 *μ*M QCT and NAC treatment prior to OGD/R treatment.

### 3.2. Low Concentration of QCT Inhibited OGD/R-Induced Apoptosis in H9c2 Cells

**Figure 2:**
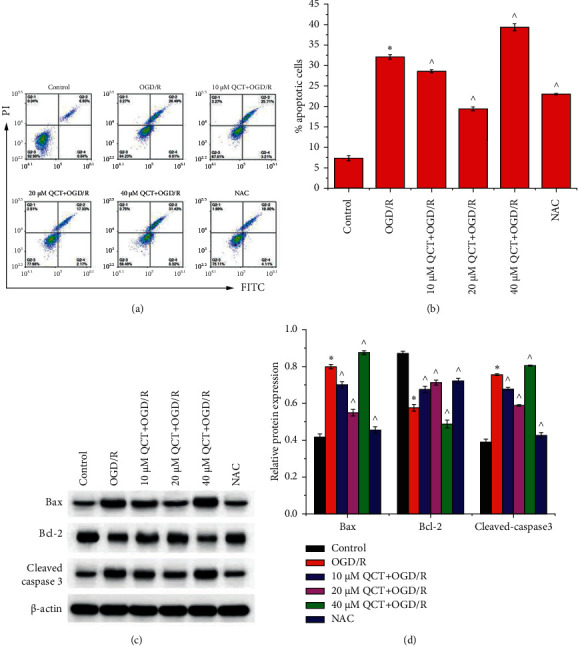
Low concentration of QCT inhibits OGD/R-induced apoptosis in H9c2 cells. (a) Apoptosis and (b) apoptosis-related protein expression in H9c2 cells. Cells were cultured with different concentrations of QCT or NAC and subsequently cultured for 6 h in glucose-free DMEM (TBD) at 37°C with 5% CO_2_, 5% O_2_, and 90% N_2_; thereafter, the cells were cultured for 24 h in DMEM (HyClone) supplemented with 10% FBS (Gibco) and maintained at 37°C with 5% CO_2_ and 95% air. Data were presented as the mean ± SD, *n* = 3, ^*∗*^*p* < 0.05 vs. the control group, and ^*p* < 0.05 vs. the OGD/R group.

Flow cytometry showed that the OGD/R-induced apoptosis of H9c2 cells was aggravated with 40 *μ*M QCT treatment and alleviated with 10 and 20 *μ*M QCT and NAC treatment prior to OGD/R treatment (Figure 2(a)). Western blotting demonstrated that OGD/R treatment increased Bax and cleaved caspase-3 expression and decreased Bcl-2 expression (Figure 2(b)). The effects of OGD/R were aggravated with 40 *μ*M QCT treatment and mitigated with 10 and 20 *μ*M QCT and NAC treatment prior to OGD/R treatment.

### 3.3. Low Concentration of QCT Promoted Mitochondrial Function in H9c2 Cells Subjected to OGD/R

**Figure 3:**
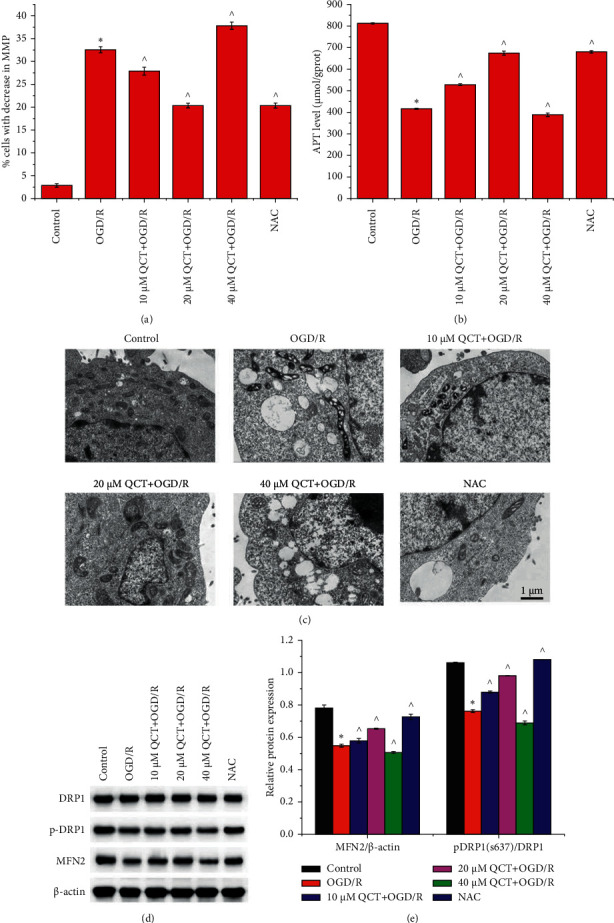
Low concentration of QCT promotes mitochondrial function in H9c2 cells subjected to OGD/R. The (a) proportion of H9c2 cells with decreased MMP and (b) ATP levels, as well as (c) mitochondrial ultrastructure- and (d) mitochondrial function-related protein expression. Cells were cultured with different concentrations of QCT or NAC and subsequently cultured for 6 h in glucose-free DMEM (TBD) at 37°C with 5% CO_2_, 5% O_2_, and 90% N_2_; thereafter, the cells were cultured for 24 h in DMEM (HyClone) supplemented with 10% FBS (Gibco) and maintained at 37°C with 5% CO_2_ and 95% air. Data were presented as the mean ± SD, *n* = 3, ^*∗*^*p* < 0.05 vs. the control group, and ^*p* < 0.05 vs. the OGD/R group.

As shown in Figure 3, OGD/R treatment increased the proportion of H9c2 cells with decreased MMP (Figure 3(a)), reduced ATP levels (Figure 3(b)), and induced ridge structure deformation (Figure 3(c)). The effects of OGD/R were aggravated with 40 *μ*M QCT treatment and mitigated with 10 and 20 *μ*M QCT and NAC treatment prior to OGD/R treatment. OGD/R treatment induced MFN2 expression, and DRP1 (S637) phosphorylation inhibition was aggravated with 40 *μ*M QCT treatment and alleviated with 10 and 20 *μ*M QCT and NAC treatment prior to OGD/R treatment (Figure 3(d)).

### 3.4. Low Concentration of QCT Inhibited ERK1/2 Signaling Activation Induced by OGD/R in H9c2 Cells

**Figure 4:**
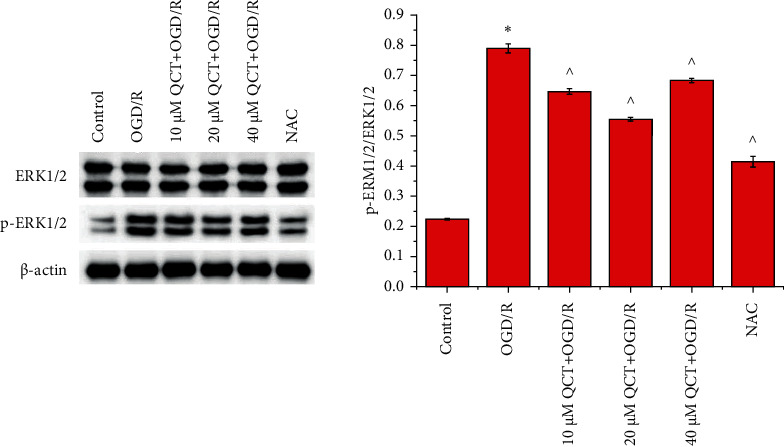
Low concentration of QCT inhibits ERK1/2 signaling activation in H9c2 cells subjected to OGD/R. ERK1/2 and p-ERK1/2 expression in H9c2 cells. Cells were cultured with different concentrations of QCT or NAC and subsequently cultured for 6 h in glucose-free DMEM (TBD) at 37°C with 5% CO_2_, 5% O_2_, and 90% N_2_; thereafter, the cells were cultured for 24 h in DMEM (HyClone) supplemented with 10% FBS (Gibco) and maintained at 37°C with 5% CO_2_ and 95% air. Data were presented as the mean ± SD, *n* = 3, ^*∗*^*p* < 0.05 vs. the control group, and ^*p* < 0.05 vs. the OGD/R group.

As shown in Figure 4, OGD/R treatment inactivated ERK1/2 signaling activation. The effect was mitigated by treatment with 10 and 20 *μ*M QCT and NAC prior to OGD/R treatment.

## 4. Discussion

Numerous studies have shown that the primary pathological mechanisms of I/R injury are intracellular calcium overload-induced cell injury and oxidative stress injury caused by excessive generation of free radicals, in addition to some other potential processes, including microvascular injury, inflammation, and endothelial dysfunction. Among these causes, apoptosis and oxidative stress play essential roles in I/R injury [8, 20, 21]. Oxidative stress is characterized by an imbalance between the elimination and generation of free radicals in response to inadequate antioxidant defenses or increased ROS generation [22]. The process of ischemia-reperfusion leads to a large amount of ROS generation from mitochondria [23, 24], thereby resulting in the opening of the mitochondrial permeability transition pore, ultimately leading to I/R injury [25]. The structure and function of the mitochondrial permeability transition pore are closely associated with the biological functions of cells. Under normal conditions, the mitochondrial permeability transition pore opens and closes intermittently, which is conducive to the balance of calcium ions [26]. However, oxidative stress contributes to the opening of the mitochondrial permeability transition pore, leading to the disruption of ATP production and aggregation of protein macromolecules, subsequently resulting in osmotic swelling of the mitochondrial matrix and functional impairment [27–29]. In addition, continuous opening of the mitochondrial permeability transition pore contributes to the decrease in MMP and the release of cytochrome C into the cytosol [30], leading to the formation of caspase-9, which accelerates the proteolytic cleavage of intracellular apoptosis-related proteins, such as caspase-3, ultimately inducing apoptosis [31–34]. A decrease in MMP is associated with mitochondrial dysfunction, which in turn causes ROS generation and initiates mitochondria-mediated apoptotic signaling [35]. Moreover, ROS are involved in the regulation of Bcl-2 family proteins, a mitochondrial channel that modulates the mitochondrial permeability transition pore activity [36, 37]. QCT, as a member of the flavonoid family with antioxidant effects, is known to have vascular protection and anticancer and anti-inflammatory effects. Quercetin induces bidirectional, hormonal, and dose-dependent effects, acting as an antioxidant for chemoprophylaxis at low concentrations, but as a prooxidant for chemotherapy at high concentrations [38]. This work suggested that low concentrations of QCT alleviated oxidative stress by decreasing ROS generation, MDA levels, and SOD levels, decreased apoptosis by regulating apoptosis-related proteins, such as Bax, Bel-2, and cleaved caspase-3, reduced the proportion of cells with decreased MMP, enhanced ATP production, and improved the mitochondrial structure in H9c2 cells subjected to OGD/R. These effects were similar to those of NAC, a ROS inhibitor.

Oxidative stress can also be involved in modulating mitochondrial fission and fusion by regulating mitochondrial fission-associated proteins, such as DRP1, and mitochondrial fusion-associated proteins such as MFN2, thereby disturbing energy metabolism and ultimately mitochondrial apoptosis [39–41]. Mitochondrial fission- and fusion-related proteins are regulated by numerous molecular mechanisms. A previous study indicated that p-ERK1/2 inhibition promotes the phosphorylation of DRP1 at S637 [42]. However, the phosphorylation of ERK1/2 is regulated by ROS [43]. This study demonstrated that low concentrations of QCT inhibited the phosphorylation of ERK1/2 and increased MFN2 expression and the phosphorylation of DRP1 at S637 in H9c2 cells subjected to OGD/R.

## 5. Conclusion

Collectively, our results showed that high concentrations of QCT (more than 40 *μ*M) were cytotoxic to H9c2 cells subjected to OGD/R. Low concentrations of QCT (less than 20 *μ*M) alleviated OGD/R-induced injury to H9c2 cells by suppressing oxidative stress and mitochondrial dysfunction-regulated apoptosis. The mitochondrial apoptosis mechanism is partially mediated via the ERK1/2-DRP1 (S637) signaling pathway. The present study indicated that QCT might be a potential candidate for I/R injury prevention.

## Data Availability

The data used to support the findings of this study are included within the article.
